# Urachal Villous Adenoma Coexistent With Urachal Carcinoma: A Case Report

**DOI:** 10.1002/iju5.70035

**Published:** 2025-04-29

**Authors:** Shotaro Hatano, Yousuke Shimizu, Hirotake Fujii, Koken Hayashi, Ken Maekawa, Yasuyuki Miyauchi, Takaki Sakurai, Kenji Mitsumori, Hiroyuki Onishi

**Affiliations:** ^1^ Department of Urology Osaka Red Cross Hospital Osaka Japan; ^2^ Department of Pathology Osaka Red Cross Hospital Osaka Japan

**Keywords:** adenocarcinoma, partial cystectomy, urachus, urinary tract, villous adenoma

## Abstract

**Introduction:**

Urinary tract villous adenomas are uncommon, urachal villous adenomas being especially rare. While their malignant potential remains uncertain, villous adenomas may have malignant components. Here, we present a case of the coexistence of a urachal villous adenoma and urachal carcinoma.

**Case Presentation:**

We report the case of an 86‐year‐old woman with a urachal tumor of the bladder wall. A biopsy yielded a diagnosis of villous adenoma. Because of the possibility of coexisting malignancy, we performed a partial cystectomy, including excision of the umbilical ligament. Examination of the operative specimen revealed mucinous adenocarcinoma. She has remained recurrence‐free for 8 months postoperatively.

**Conclusion:**

We here report the coexistence of a villous adenoma and mucinous adenocarcinoma of the urachus. Because urachal villous adenomas can have malignant components, they require aggressive treatment. This rare combination should be kept in mind.

**Summary**

- We here report a patient with coexisting urachal villous adenoma and urachal carcinoma.
- Urachal villous adenomas are rare and can have malignant components.
- Thus, they may require aggressive treatment such as partial cystectomy, including excision of the umbilical ligament.
- This rare combination should be kept in mind whenever a villous adenoma is diagnosed.

## 1 Introduction

Villous adenomas are benign tumors that most commonly originate in intestinal epithelium [1]. Urachal villous adenomas are uncommon [2]. While their malignant potential has not been fully established, it is known that urinary tract villous adenomas may have malignant components [1]. Here, we present a case of the coexistence of urachal villous adenoma and mucinous adenocarcinoma in the urinary bladder.

## 2 Case Presentation

**FIGURE 1:**
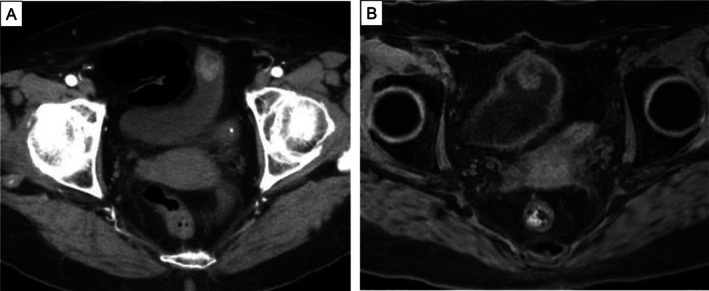
(A) Abdominal enhanced computed tomography image revealing an 18‐mm bladder tumor located on the dome of the bladder and contiguous with the umbilical ligament tumor. (B) Diffusion‐weighted magnetic resonance image showing an 18‐mm solid tumor on the dome of the bladder and exhibiting the inchworm sign.

**FIGURE 2:**
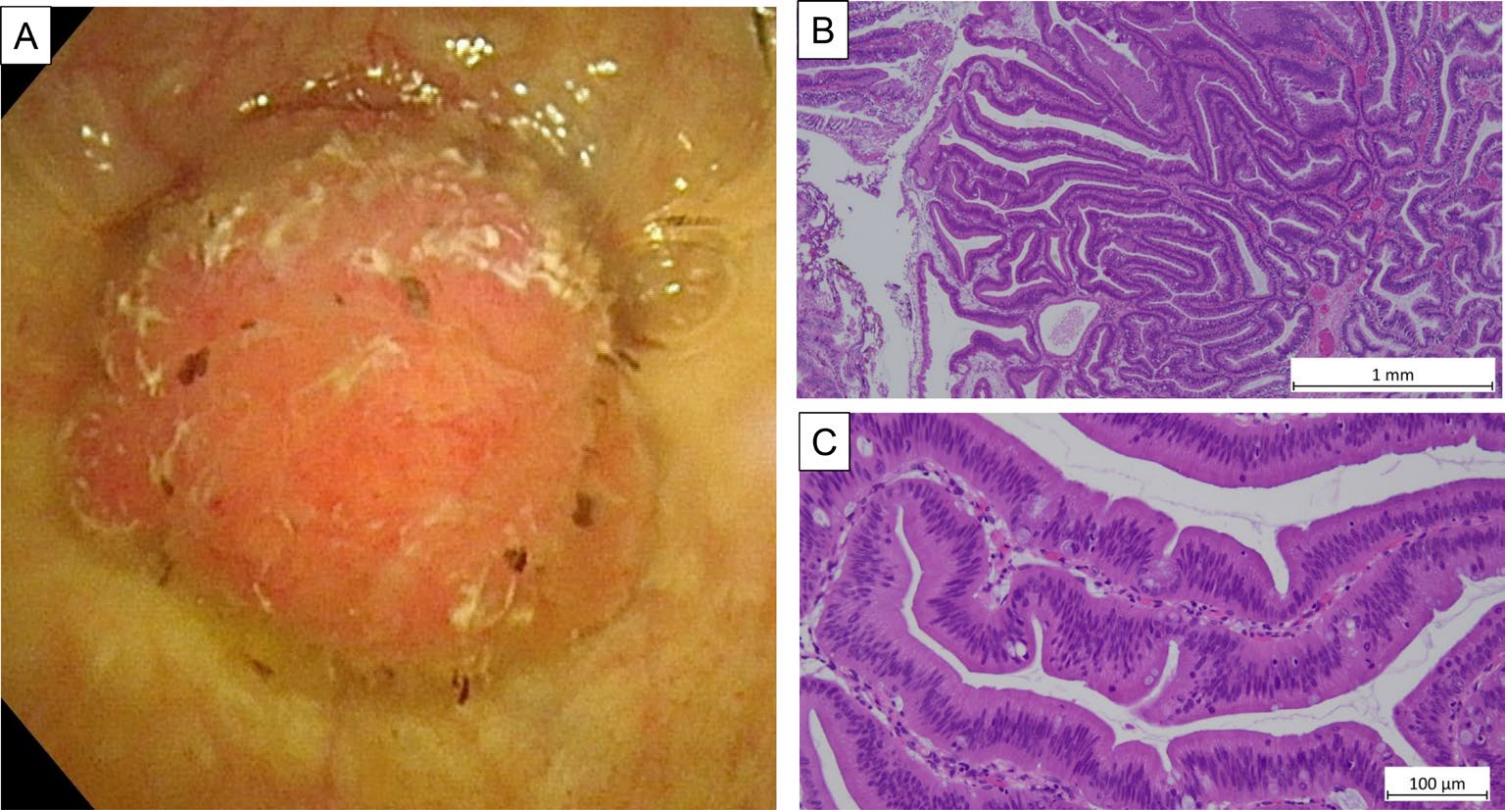
(A) Cystoscopy revealed a papillary tumor on the dome of the bladder. (B, C) Hematoxylin–eosin‐stained slides of a specimen obtained by transurethral biopsy showing a papillary tumor with foci of villous proliferation of atypical columnar epithelium with swollen spindle‐shaped, darkly stained nuclei that maintain polarity. (B) ×100, (C) ×400.

**FIGURE 3:**
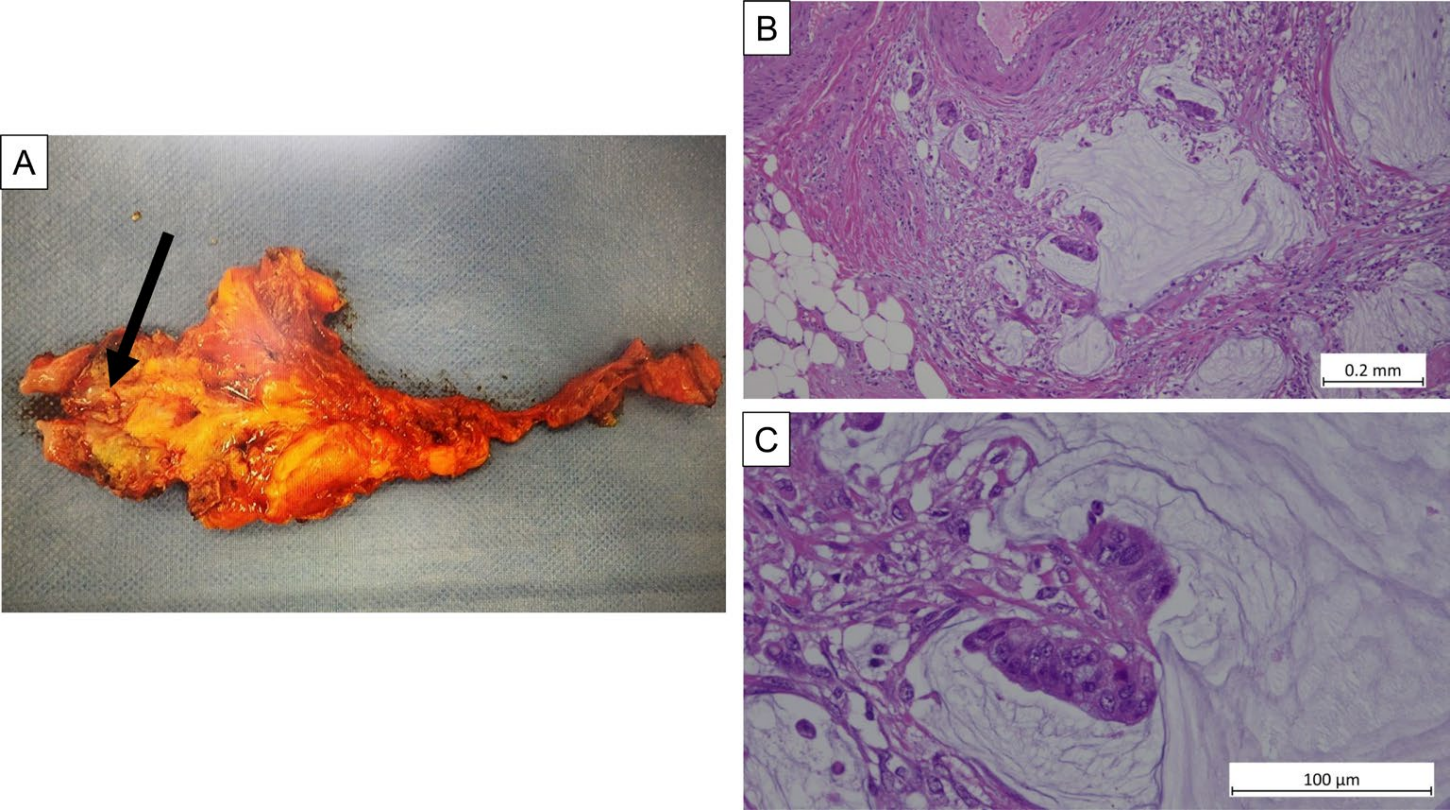
(A) Macroscopic photograph showed a mucin‐producing tumor. (B, C) Hematoxylin–eosin‐stained slide from the surgical specimen showing a papillary tumor. There is abundant mucus retention in the bladder mucosa and tissue, and mucus within the cell death zone on the border. Evidence of mucinous carcinoma includes proliferation of atypical columnar cells of irregular size with large nuclei, and floating fascicles. Final pathological diagnosis: mucinous adenocarcinoma. (B) ×100, (C) ×400.

An 86‐year‐old woman was referred to our hospital because plain computed tomography had shown a bladder tumor. An enhanced computed tomography scan revealed a tumor on the dome of the bladder and contiguous with the umbilical ligament (Figure 1A). Magnetic resonance imaging revealed a bladder tumor with the inchworm sign, but muscle invasion could not be excluded (Figure 1B). Cystoscopy showed a papillary tumor at the dome of the bladder (Figure 2A). We performed a transurethral resection, during which mucus secretion from the base of the resection was observed, suggesting a residual mucin‐producing tumor. The pathological diagnosis was villous adenoma (Figure 2B,C). While villous adenomas are benign tumors, they can have malignant components. In our patient, invasion of the muscle layer could not be ruled out by magnetic resonance imaging or operative findings. Because preoperative cystoscopy and imaging studies had shown no evidence of tumors in other areas, we decided to perform a laparoscopic partial cystectomy, including resection of the umbilical ligament. The surgery was performed under general anesthesia with the patient in the lithotomy position. The camera port was placed in a supraumbilical position and four laparoscopic ports were placed. The bladder wall was opened and the tumor excised along the transurethral marking and removed, together with the umbilical ligament. The operation took 190 min with minimal blood loss. The final pathological diagnosis was mucinous adenocarcinoma, pT3b, according to a novel staging system [3] (Figure 3). The patient recovered well and was discharged 11 days after surgery. No adjuvant chemotherapy was administered. The patient remained recurrence‐free 8 months postoperatively.

## 3 Discussion

Villous adenomas are benign tumors that most commonly originate in intestinal epithelium [1]. Thus, villous adenomas of the urinary tract are rare [2]. These lesions occur predominantly in men, most of whom are over 50 years old. Patients may present with hematuria or mucosuria and abdominal symptoms suggestive of inflammation [4].

Pure villous adenomas have a good prognosis with no postoperative recurrences. However, some patients with combinations of villous adenoma and adenocarcinoma in the urinary tract have been reported [5]. Cheng et al. reported 23 patients with villous adenoma of the urinary tract, 8 of whom had coexisting adenocarcinoma [2]. Seibel et al. reported 18 patients with villous adenomas of the urinary tract, 11 of whom had coexisting adenocarcinomas. Approximately half of urinary tract villous adenomas coexist with adenocarcinoma and/or another malignancy [6]. Villous adenomas of the urachus are very rare; only 10 cases have been reported [1, 2, 6, 7]. Data of these patients and the present case are summarized in Table 1. The patients' median age was 60 years (range 25–86 years). Four of the 11 patients were male. After the establishment of a diagnosis by transurethral resection, six patients underwent partial cystectomy and two underwent cystoprostatectomy. Three patients, including one with adenocarcinoma, received no further treatment. Six of the 11 patients had coexisting adenocarcinoma or urothelial carcinoma. These adenomas may also have malignant potential.

**TABLE 1:** Villous adenoma of the urachus in 11 cases *including* this case.

Case	Age	Sex	Pathology of TUR	Treatment	Final pathological diagnosis	Follow‐up (years)	Outcome
1	25	F	VA	Only TUR		NA	NA
2	50	M	VA	Only TUR		NA	NA
3	74	F	Neoplasm	Partial cystectomy	VA	1.1	NED
4	57	F	No TUR	Partial cystectomy	VA	3	NED
5	56	M	VA	Cystoprostatectomy	VA	NA	NA
6	66	M	VA	Partial cystectomy	VA with *urothelial carcinoma*	2	Recurrence of sarcomatoid carcinoma
7	57	F	No TUR	Partial cystectomy	VA with adenocarcinoma	NA	NA
8	73	F	VA with adenocarcinoma	Only TUR		11	NED
9	60	M	VA	Cystoprostatectomy	Mucinous adenocarcinoma	4	NED
10	78	F	VA	Partial cystectomy	VA with adenocarcinoma	NA	NA
This case	86	F	VA	Partial cystectomy	Mucinous adenocarcinoma	0.5	NED

Abbreviations: NA, not available; NED, no evidence of disease; TUR, transurethral resection; VA, villous adenoma.

Although there is no established gold standard for the treatment of urachal cancer, recent data indicate that radical cystectomy is not superior to partial cystectomy and does not improve outcomes compared to partial cystectomy [8]. Since 2010, the use of radical cystectomy has decreased in favor of partial cystectomy, and partial cystectomy would likely be the preferred surgical choice in the future [8].

Histologically, villous adenomas exhibit rounded projections of pseudostratified columnar epithelium with goblet‐type mucin‐producing cells [9]. Approximately 50% of urinary tract villous adenomas are cytokeratin 7 positive, whereas intestinal villous adenomas are usually cytokeratin 7 negative [2]. Molecular studies performed on one bladder villous adenoma showed DNA aneuploidy and strong expression of p53 [10].

The mechanism by which glandular epithelial lesions arise in the urothelial‐lined urinary tract has yet to be elucidated. There are two hypotheses regarding the pathogenesis of villous adenoma originating in the urothelium. The distal colorectum and bladder both arise from the partitioning of the cloaca by the urorectal septum in the embryo. One possibility is that cloacal nests remain in the adult bladder and urachus, and these remnants have the potential to give rise to glandular epithelial neoplasms [11]. The other theory proposed is that injured stem cells in the urothelium undergo glandular differentiation, resulting in both glandular metaplasia and neoplasia [12].

Bladder dome and the posterior wall are the most frequent sites of villous adenocarcinomas [13]. This fact supports the former hypothesis. This case is also likely to fit the former hypothesis. However, there is a case of villous adenoma in the left wall. Since the anatomical location is different from the location of the cloaca, the latter hypothesis cannot be denied.

There is a phenomenon known as “adenoma–carcinoma sequence,” which is believed to involve genetic mutations including p53 in the field of colorectal cancer [14]. It is reported that a urachal tubulovillous adenoma shares similar oncogenes and tumor suppressor proteins such as p53 with colonic tubulovillous adenoma. The progression from colonic adenoma to adenocarcinoma is genetically well characterized and thus may be applicable in urachal tumors [15]. This phenomenon could explain the coexistence and heterogeneity of adenomas and carcinomas in regard to villous adenomas of the urinary tract. However, it cannot be excluded that urachal adenoma and adenocarcinoma may in fact coexist in a solitary lesion [15]. Whatever the mechanism involved, the relatively frequent coexistence of villous adenomas and carcinoma necessitates thorough specimen collection and tumor resection whenever a biopsy reveals a urinary tract villous adenoma [6].

In the present case, we performed transurethral resection for a villous adenoma located at the dome of the bladder. After resection of the tumor, we noted mucus secretion from its base, likely indicating that the urachal tumor had not been completely resected. We therefore excised the bladder tumor together with the urachal remnant; the final diagnosis was urachal adenocarcinoma. We are carefully monitoring this patient for recurrence.

## 4 Conclusion

We here report a patient with a coexisting villous adenoma and adenocarcinoma of the urachus. A superficial biopsy failed to reveal the adenocarcinoma. Villous adenomas are very uncommon, particularly in the urachus. They can coexist with malignant components. Accordingly, they may require more aggressive treatment than other types of adenoma. This rare possibility should be kept in mind.

## Ethics Statement

The authors have nothing to report.

## Consent

We obtained written informed consent for publication of this report from the patient.

## Conflicts of Interest

The authors declare no conflicts of interest.
